# Development of hepatocellular carcinoma from various phases of chronic hepatitis B virus infection

**DOI:** 10.1371/journal.pone.0261878

**Published:** 2021-12-28

**Authors:** Takanori Suzuki, Kentaro Matsuura, Yoshihito Nagura, Etsuko Iio, Shintaro Ogawa, Kei Fujiwara, Shunsuke Nojiri, Hiromi Kataoka, Yasuhito Tanaka

**Affiliations:** 1 Department of Gastroenterology and Metabolism, Nagoya City University Graduate School of Medical Sciences, Nagoya, Japan; 2 Department of Virology and Liver Unit, Nagoya City University Graduate School of Medical Sciences, Nagoya, Japan; 3 Department of Gastroenterology and Hepatology, Faculty of Life Sciences, Kumamoto University, Kumamoto, Japan; Nihon University School of Medicine, JAPAN

## Abstract

**Background & aims:**

There is insufficient data on the clinical course of chronic hepatitis B (CHB) patients in the immune-tolerant (IT) and immune-clearance, inactive (IC) phases over a long follow-up period.

**Design:**

We enrolled 466 CHB patients from our historical cohort, including 56 IT+MA　 (mildly active), 134 IC, 230 with chronic active hepatitis (CH) and 46 with liver cirrhosis (LC), who were categorized to each phase by at least one year of follow-up period from the first visit to our hospital. We investigated long-term risks, and their factors, of developing hepatocellular carcinoma (HCC), and the transition between the clinical phases, especially in the IT+MA and IC groups.

**Results:**

Of the 56 patients in the IT+MA group, 27 remained the IT+MA phase, but 29 transitioned to the CH phase and started nucleot(s)ide analogue (NA) treatment during the follow-up period. Meanwhile, of the 134 patients in the IC group, only 5 started NA treatment after progressing to the CH phase. The development of HCC from the IT+MA, IC, CH, and LC groups was observed in 2, 2, 9, and 20 cases, respectively. The cumulative incidence rates of developing HCC in the IT+MA, IC, CH, and LC groups were 9.9, 1.8, 3.0, and 53.1% at 10 years. In the CH and LC group, patients who developed HCC were older, had higher levels of FIB-4 index, M2BPGi, HBcrAg and AFP, and had lower levels of albumin and platelet counts. In CH patients, FIB-4 index levels were elevated at the diagnosis of HCC compared to baseline, whereas these decreased during the follow-up period in non-HCC patients.

**Conclusions:**

HCC occurred at a certain rate among patients in the IT+MA and IC groups. Careful follow-up is required for CH patients with higher levels of FIB-4 index and/or M2BPGi because of the high incidence of HCC development. (299 words)

## Introduction

Chronic hepatitis B (CHB), caused by persistent hepatitis B virus (HBV) infection, affects approximately 260 million individuals worldwide [1]. Although most CHB patients have promising clinical outcomes, an estimated 15–40% of patients develop cirrhosis and/or hepatocellular carcinoma (HCC) [2]. In Asia, hepatitis B is mostly transmitted perinatally or during infancy. After infection with HBV, the earliest phase is the immune tolerant (IT) phase, which is characterized by very high viral replication, hepatitis B e antigen (HBeAg) positivity, and normal or minimally elevated serum alanine aminotransferase (ALT) levels [3]. Patients in the IT phase are mostly younger than thirty years [4, 5]. In the immune-clearance phase, patients exhibit hepatitis activity or episodic flares during adolescence or adulthood. These events might cause fibrosis or cirrhosis during the HBeAg positive phase, but may lead to declining HBV DNA levels and HBeAg seroconversion. After HBeAg seroconversion, most patients enter the inactive phase with low HBV DNA and normal ALT [6]. However, in the inactive phase, reactivation of HBV with either HBeAg seroreversion or the development of precore or basal core promoter mutation sometimes occurs and causes hepatitis.

In the IT phase, considering the minimal risk of disease progression, the anticipated long-term treatment of younger patients and, potentially, a poor response to treatment, nucleiot(s)ide analogue (NA) treatment is generally not recommended [7]. However, a Korean study showed that the risk of developing HCC was higher for untreated patients in the IT phase than for patients the in immune active phase on NA therapy [8]. In contrast, another study showed that the cumulative risk of HCC was similar for patients in the IT phase and those with a virological response to antivirals [9]. One possible reason for the discrepancy between these results might be attributable to the difficulty of the definition of the IT phase.

Inactive carriers (IC) form a large group of those infected with HBV and the prognosis of IC usually is benign. Long-term follow up of IC patients has indicated that the vast majority have a low risk of cirrhosis and/or HCC [6]. Furthermore, although the IT and IC phases are considered to have a good prognosis, hepatitis could develop in these phases over a long period [10].

Although many previous studies showed that treatment of CHB with NAs reduced the rate of development of HCC [11–13], Hosaka et al. reported that the cumulative incidence rates of developing HCC at 5 years were 3.7% and 13.7% for the entecavir and control groups [12]. The most significant risk factor for developing HCC during NA treatment is advanced hepatic fibrosis [14]. The well-known markers for hepatic fibrosis, fibrosis-4 (FIB-4) index and M2BPGi, can predict likely the development of HCC in CHB patients under NA therapy [15, 16].

On this basis, we investigated retrospectively the long-term risks, and their factors, of developing HCC, and the transition of the clinical phase in chronically HBV-infected patients, especially in the IT and IC phases, compared with those with chronic hepatitis (CH) and liver cirrhosis (LC).

## Methods

### Patients and study design

**Fig 1:**
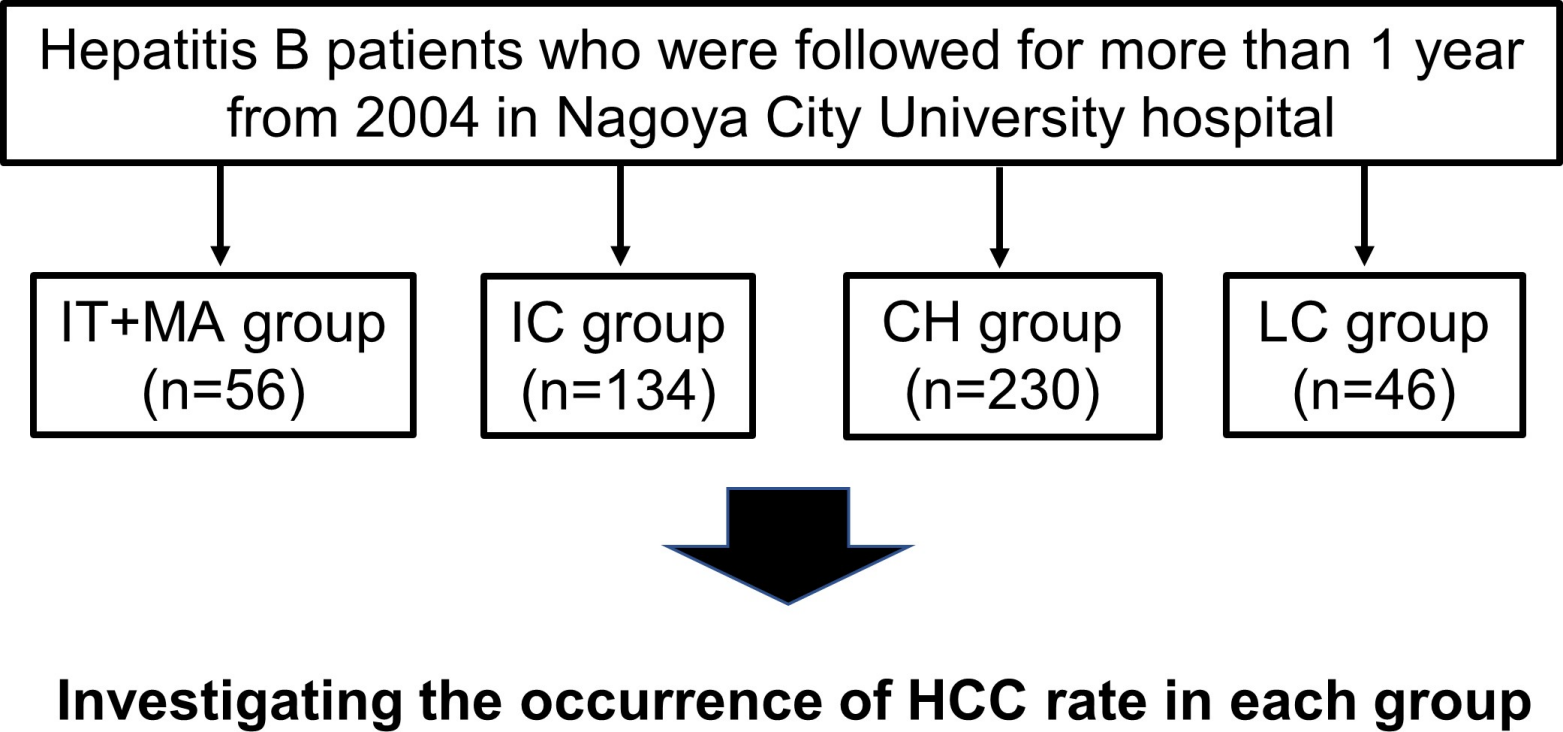
Patient flow chart. Abbreviations: IT+MA, immune tolerant + mildly active, IC, inactive carrier; CH, chronic active hepatitis; LC, liver cirrhosis; HCC, hepatocellular carcinoma.

The study subjects were recruited from a historical cohort of 466 chronically HBV-infected patients, which included 56 IT+MA (mildly active) phase, 134 IC, 230 CH (chronic active hepatitis), and 46 LC patients. They were followed for more than one year from 2004 in Nagoya City University hospital (Fig 1). Our study defined the IT+MA phase as follows: being HBeAg positive, HBV DNA >20,000 IU/ml, and ALT <2×upper limits of normal (ULN) referring to the previous study [8], which is recommended for monitoring and assessment of fibrosis in the Asian Pacific Association for the Study of the liver (APASL) guideline. The IC phase was defined according to the criteria of the APASL as follows: being HBeAg negative, hepatitis B e antibody (anti-HBe) positive, HBV DNA <2,000 IU/ml, and ALT <ULN [4]. Non-cirrhotic patients who were treated with NAs for more than one year were defined as CH, and the LC group was defined comprehensively as the presence of the following criteria: coarse liver echotexture or nodular liver surface on ultrasonography, clinical features of portal hypertension, including splenomegaly, ascites, esophageal varices and/or gastric varices, based on imaging tests including magnetic resonance imaging (MRI), computerized tomography (CT) and/or ultrasonography, or thrombocytopenia. All CH and LC patients were treated with NAs: lamivudine, entecavir, tenofovir disoproxil fumarate or tenofovir alafenamide. The following patients were excluded from this study: those co-infected with hepatitis C virus or human immunodeficiency virus, with a history of HCC and organ transplantation, or developing HCC within one year of the first visit. The normal ALT level was defined as <40 U/ml, according to the criteria of the APASL [4].

### Ethical standards

Written informed consent was obtained from each patient. The study protocol was approved by the Institutional Review Board of Nagoya City University and implemented according to the Declaration of Helsinki.

### Outcomes and follow-up evaluation

The outcome of interest was the occurrence of HCC. The start of the follow-up period was the date of the first visit to our hospital and the end was the date of diagnosing HCC or the last visit of non-HCC patients. The patients in the IT+MA and IC phases who started NA treatment during follow-up were censored when they were treated with NAs for the first time. All patients who started NA treatment were advised to continue that treatment until hepatitis B surface antigen (HBsAg) seroclearance or they met the Japanese stop criteria: HBsAg <80 IU/ml and hepatitis B core-related antigen (HBcrAg) <3 logU/ml [17, 18]. The study patients had regular clinical assessment, including HBeAg status, ALT and HBV DNA levels around every 3–6 months. For HCC surveillance, patients underwent ultrasonography, CT and/or MRI at baseline and around every 6 months thereafter. Serum α-fetoprotein levels were measured around every 3–6 months. The surveillance protocols were in accordance with the standard of care in Japan. If HCC was suspected from the screening, additional examinations, such as dynamic CT, dynamic MRI, contrast-enhanced ultrasonography and hepatic angiography, were performed as necessary. In these examinations, HCC was diagnosed by the finding of typical hypervascular feature. If no typical feature of HCC was observed, a tumor biopsy was performed to diagnose HCC.

### Laboratory and histological tests

Hematologic and blood chemistry tests were carried out using standard assays. Serum HBsAg was assessed using fully automated CLEIAs: the high-sensitivity HBsAg assay, HBsAg-HQ (Fujirebio, Inc., Tokyo, Japan) or the conventional HBsAg assay, HISCL HBsAg (Sysmex Corporation, Kobe, Japan). The seropositivity values were 0.005 IU/mL for HBsAg-HQ and 0.03 IU/mL for HISCL HBsAg. Serum HBeAg was measured using automated CLEIA system (HISCL HBeAg; Sysmex Corporation, Kobe, Japan) with a cut of index of 1.0. Serum HBV DNA levels were measured by the COBAS AmpliPrep/COBAS TaqMan HBV v2.0 assay (Roche Diagnostics K.K., Tokyo, Japan) until 2017 and, thereafter, by the Cobas AmpliPrep/Cobas TaqMan HBV Test, v2.0 (Roche Diagnostics K.K., Tokyo, Japan), according to the manufacturer’s instructions. The lower and upper limits of quantitation for the tests were 2.1 log copies/mL and 9.0 log copies/ml, and 1.3 log IU/mL and 8.2 log IU/ml, respectively. The unit change formula is “log IU/ml = log copies/ml -0.76” according to the manufacturer’s instructions. Serum M2BPGi levels were measured using HISCL M2BPGi (Sysmex Corporation, Kobe, Japan). Serum HBcrAg levels were measured using Lumipulse G HBcrAg (Fujirebio, Inc., Tokyo, Japan). The histological hepatic fibrosis index, Fibrosis-4 (FIB-4) index, was calculated as described previously: FIB-4 = (age [year] × aspartate aminotransferase (AST) [U/L]) / (platelet count [10^9^/L] × √ALT [U/L]) [19].

### Statistical analysis

All patients who met the eligibility criteria were included in the analysis. The baseline characteristics of the patients were compared using the chi-square test for categorical variables. The Mann-Whitney U test or the Kruskal-Wallis test was used to compare continuous variables. Cumulative incidence curves for HCC were estimated using the Kaplan-Meier Method.

All reported *P*-values are two sided and *P*-values of <0.05 are considered significant. EZR (Easy R, Saitama Medical Center. Jichi Medical University, Saitama, Japan), which is a modified version of R commander (version 4.0.3), was used for statistical analysis [20].

## Results

### Characteristics of study population

The baseline characteristics of the 466 patients, comprising 56 in the IT+MA phase, 134 IC, 230 CH, and 46 LC are shown in Table 1. The proportion of males was lower in the IC than in the other groups. Patients in the IT+MA group were the youngest, whereas those in the LC group were the oldest. HBV DNA levels were the highest in the IT group and the lowest in the IC group. The levels of ALT were higher in the CH and LC groups than the IT+MA and IC groups. Platelet counts were the lowest in the LC group. Few patients in the IC group had undergone IFN therapy. The median observation periods were 54, 91, 101 and 91 months for the IT+MA, IC, CH and LC groups, respectively.

**Table 1:** Baseline clinical characteristics of the patients in the various phases of chronic hepatitis B virus infection.

Phases of chronic HBV infection					
Characteristic	IT+MA	IC	CH	LC	
**Number**	**56**	**134**	**230**	**46**	***P*-value**
**Age, years**	**33 (27**–**36)**	**48 (37**–**59)**	**43 (37**–**52)**	**54 (46**–**59)**	**<0.001**
**Male gender, n (%)**	**31 (55%)**	**55 (41%)**	**150 (65%)**	**28 (61%)**	**<0.001**
**HBV genotype A / B / C / N.D., n**	**2 / 4 / 39 / 11**	**6 / 14 / 50 / 64**	**8 / 18 / 172 / 32**	**0 / 3 / 37 / 6**	**0.048**
**HBV-DNA, log**_**10**_ **IU/ml**	**8.0 (7.0**–**8.1)**	**2.8 (1.9**–**2.9)**	**6.1 (4.8**–**7.3)**	**5.8 (4.4**–**6.8)**	**<0.001**
**AST, U/l**	**28 (20**–**32)**	**21 (18**–**24)**	**66 (37**–**129)**	**68 (44**–**125)**	**<0.001**
**ALT, U/l**	**35 (22**–**46)**	**19 (14**–**23)**	**99 (45**–**223)**	**69 (41**–**153)**	**<0.001**
**Albumin, g/dl**	**4.5 (4.2**–**4.8)**	**4.6 (4.3**–**4.8)**	**4.3 (4.1**–**4.6)**	**3.9 (3.1**–**4.4)**	**<0.001**
**Total bilirubin, mg/dl**	**0.7 (0.5**–**0.9)**	**0.7 (0.6**–**0.9)**	**0.8 (0.6**–**1.0)**	**1.0 (0.8**–**1.9)**	**<0.001**
**Platelets, ×10** ^**4**^ **/dl**	**21.5 (18.4**–**25.3)**	**21.0 (18.0**–**23.8)**	**17.6 (14.6**–**21.7)**	**8.3 (6.5**–**10.4)**	**<0.001**
**FIB-4 index**	**0.64 (0.53**–**0.83)**	**1.12 (0.78**–**1.42)**	**1.66 (1.15**–**2.47)**	**4.80 (3.49**–**8.75)**	**<0.001**
**HBsAg, IU/ml**	**2,000 (2,000**–**10,000)**	**1,575 (461**–**2,000)**	**2,840 (1,290**–**10,463)**	**1,438 (459**–**2,206)**	**<0.001**
**HBeAg positive, n (%)**	**56 (100%)**	**0 (0%)**	**123 (53%)**	**25 (54%)**	**<0.001**
**IFN use, n (%)**	**14 (25%)**	**2 (1%)**	**64 (28%)**	**9 (20%)**	**<0.001**
**Duration of follow up period, month**	**54 (31**–**96)**	**91 (42**–**133)**	**101 (52**–**156)**	**105 (48**–**166)**	**<0.001**

Data from all patients are expressed as numbers for categorical data and medians (first–third quartiles) for noncategorical data.

Categorical variables were compared between groups using the chi square test, and noncategorical variables were compared using the Kruskal-Wallis test. Abbreviations: IT+MA, immune tolerant + mildly active; IC, inactive carrier; CH, chronic active hepatitis; LC, liver cirrhosis; N.D., not determined; HBV, hepatitis B virus; AST, aspartate transaminase; ALT, alanine transaminase; FIB-4, fibrosis-4; HBsAg, hepatitis B surface antigen; HBeAg, hepatitis B e antigen; IFN, interferon.

### Transition of the clinical phase from IT+MA and IC to CH

Of the 56 patients in the IT+MA group, 27 remained the IT+MA phase, but 29 progressed to the CH phase and started NA treatment during the follow-up period. The median period from baseline to the CH phase was 46 months, while the observation period for the patients who remained in the IT+MA phase was 65 months. Comparing the groups with and without NA treatment, the patients treated with NAs were younger and had higher levels of AST and albumin (Table 2). Of the 134 patients in the IC group, only 5 (4%) started NA treatment after progressing to the CH phase (n = 2), for prevention of HBV reactivation under immunosuppressive therapy (n = 2) and after developing HCC (n = 1).

**Table 2:** Baseline clinical characteristics of the patients in the IT+MA phase, according to the transition of clinical phase thereafter.

Baseline characteristic of the study patients			
**Characteristic**	**IT+MA→CH**	**IT+MA→IT+MA**	
**Number**	**29**	**27**	***P*-value**
**Age, years**	**36 (31**–**48)**	**48 (37**–**59)**	**<0.001**
**Male gender, n (%)**	**17 (59%)**	**14 (52%)**	**N.S.**
**HBV DNA, log**_**10**_ **IU/ml**	**7.9 (6.6**–**8.2)**	**7.9 (7.4**–**8.3)**	**N.S.**
**AST, U/l**	**30 (26**–**34)**	**22 (18**–**31)**	**0.006**
**ALT, U/l**	**40 (29**–**51)**	**24 (18**–**45)**	**N.S.**
**Albumin, g/dl**	**4.7 (4.4**–**4.9)**	**4.4 (4.1**–**4.6)**	**0.024**
**Total bilirubin, mg/dl**	**0.8 (0.4**–**1.0)**	**0.6 (0.5**–**0.8)**	**N.S.**
**Platelets, ×10** ^**4**^ **/dl**	**23.6 (19.9**–**26.0)**	**19.3 (18.4**–**23.4)**	**N.S.**
**FIB-4 index**	**0.73 (0.56**–**0.93)**	**0.64 (0.43**–**1.49)**	**N.S.**
**HBsAg, IU/ml**	**2,000 (2,000**–**3,212)**	**2,000 (2,000**–**8,015)**	**N.S.**

Data from all patients are expressed as medians (first–third quartiles) for noncategorical data. Noncategorical variables were compared using the Mann-Whitney U test. Abbreviations: IT+MA, immune tolerant + mildly active; CH, chronic active hepatitis; N.S., not significant; HBV, hepatitis B virus; AST, aspartate transaminase; ALT, alanine transaminase; FIB-4, fibrosis-4; HBsAg, hepatitis B surface antigen.

### Clinical events

**Fig 2:**
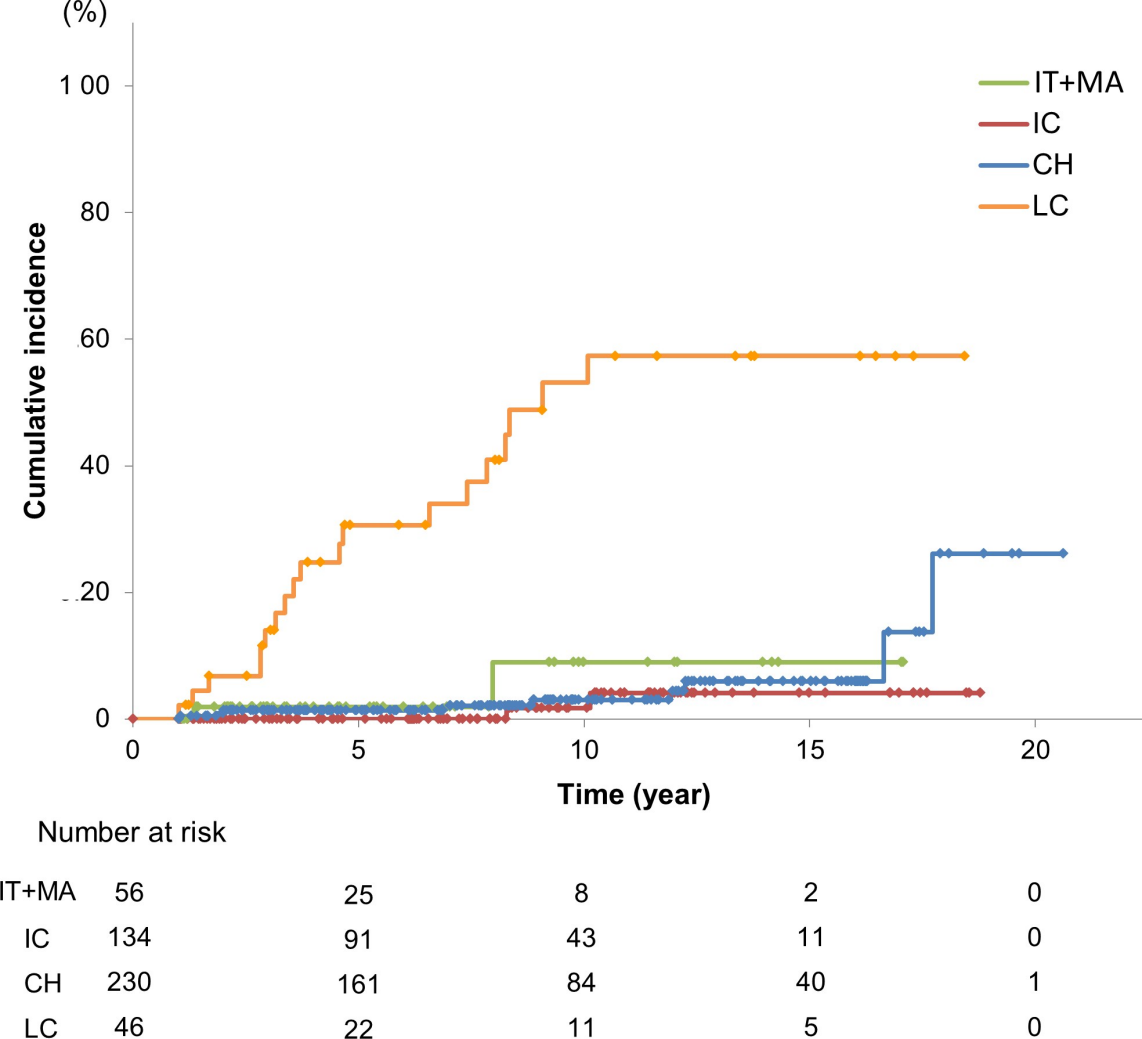
Cumulative incidence of hepatocellular carcinoma in patients in the various phases of chronic hepatitis B virus infection. The cumulative incidence curves for hepatocellular carcinoma were estimated using the Kaplan-Meier method. Abbreviations: IT+MA, immune tolerant + mildly active, IC, inactive carrier; CH, chronic active hepatitis; LC, liver cirrhosis; HCC, hepatocellular carcinoma.

Of the 56 IT+MA phase, 134 IC, 230 CH, and 46 LC patients, the development of HCC was observed in 2, 2, 9, and 20, respectively. The cumulative incidence rates of developing HCC in the IT, IC, CH, and LC groups were 1.9, 0, 1.4, and 30.6% at 5 years, 9.9, 1.8, 3.0, and 53.1% at 10 years, respectively (Fig 2).

Interferon (IFN) therapy is known to suppress hepatic carcinogenesis and improve the prognosis of CHB patients [21]. Therefore, the patients who underwent IFN treatment were excluded, and we monitored the development of HCC among 42 IT+MA, 132 IC, 166 CH, and 37 LC patients (S1 Table). The cumulative rates of developing HCC in the IT+MA, IC, CH, and LC groups were 2.6, 0, 1.9, and 49.7% at 5 years and 2.6, 2.0, 4.4, and 74.5% at 10 years, respectively (S1 Fig). Thus, the trend was similar among all patients, with or without past IFN therapy.

### The cases developing hepatocellular carcinoma from the groups in the IT+MA and IC phases

As mentioned above, the development of HCC was observed in two patients in each of the IT+MA and IC group. These cases are reported in detail:

The IT+MA-HCC case 1 was 61 years old at his first visit, he was classified as in the IT+MA phase, and developed HCC 1.5 years after his first visit at the age of 62. His clinical data at diagnosis of HCC showed HBV genotype C, HBeAg positivity, HBV DNA 5.4 log IU/ml, ALT 52 U/ml, FIB-4 index 3.7. His FIB-4 index remained high until he developed HCC (S2 Fig). The IT+MA -HCC case 2 was 48 years old at his first visit, he was classified as in the IT+MA phase, and developed HCC 8 years after his first visit at the age of 56. His clinical data at diagnosis of HCC showed HBV genotype C, HBeAg positivity, HBV DNA 6.2 log IU/ml, ALT 59 U/ml, FIB-4 index 4.2. His FIB-4 index was also high at the time of developing HCC.

The IC-HCC case 1 was 65 years old at his first visit, he was classified as in the IC group and developed HCC 8.2 years after his first visit at the age of 73. His clinical data at baseline showed HBV genotype C, HBeAg negativity, HBV DNA 4.0 log IU/ml, ALT 8 U/l, FIB-4 index 3.8. His FIB-4 index had remained high until he developed HCC (S3 Fig). The IC-HCC case 2 was 60 years old at his first visit, he was classified in the IC group and developed HCC 10.1 years after his first visit at the age of 71. His clinical data at baseline showed the HBV genotype was not determined, HBeAg negativity, HBV DNA 1.3 log IU/ml, ALT 36 U/l, FIB-4 index 2.3.

### Risk factors for developing hepatocellular carcinoma from the phases of chronic hepatitis and liver cirrhosis under treatment with NAs

**Fig 3:**
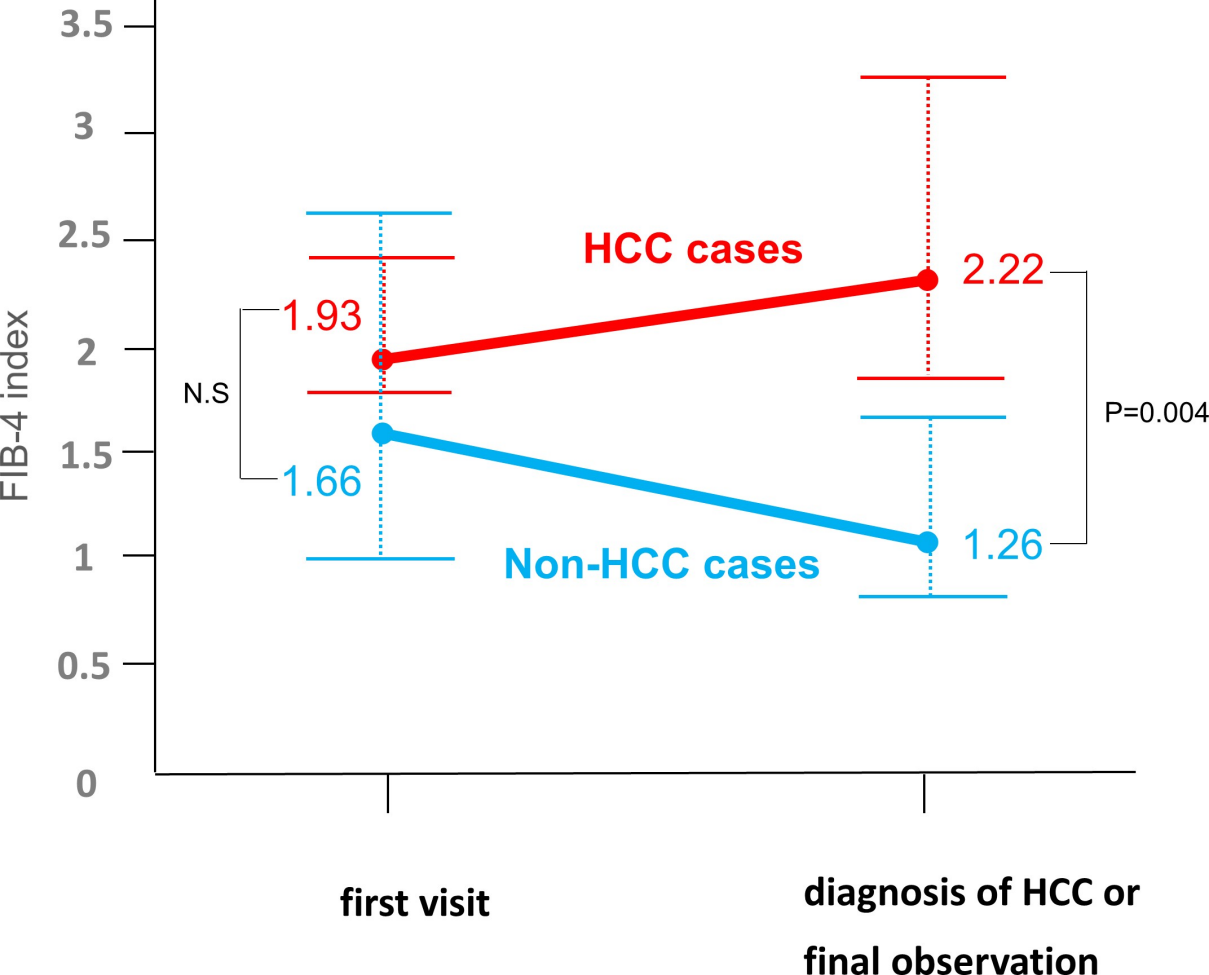
Transition of FIB4-index in CH group. We depict the transition of FIB-4 index levels from the first visit to our hospital to the diagnosis of hepatocellular carcinoma (HCC) or the last follow-up visits of patients who did not develop HCC. Red line and numbers represent mean FIB-4 index levels in the HCC cases, and those in blue represent in the non-HCC cases. FIB-4 index levels were compared between the HCC and the non-HCC groups at the first visit and the diagnosis of HCC or the last follow-up visits using the Mann-Whitney U test. Above the horizonal line represents third quantile, and below the horizonal line represents first quantile. Abbreviations: FIB-4, fibrosis-4; CH, chronic active hepatitis; HCC, hepatocellular carcinoma.

Previous studies indicated that the major risk factors for developing HCC under NA treatment are advanced hepatic fibrosis and age [22, 23]. Our results showed that the cumulative incidence rate of developing HCC was higher in the LC than the CH group. When we examined the patients under NA treatment, namely the CH and LC groups, the patients who developed HCC were older, had higher levels of FIB-4 index, M2BPGi, and AFP, and had lower levels of albumin and platelet counts (S2 Table), consistent with the previous findings, mentioned above. Nine CH cases developed HCC, the median age at the diagnosis of HCC was great, 64 years. In these patients, FIB-4 index levels were elevated at the diagnosis of HCC, compared to baseline, whereas these decreased during the follow-up period in non-HCC patients. As a result, FIB-4 index levels were higher in HCC patients at the diagnosis of HCC than in non-HCC patients at the last follow-up visits (*P* = 0.004) (Fig 3). Serum M2BPGi levels were measured in 147 of the 230 CH cases at the last follow-up visit, revealing that the M2BPGi levels at baseline did not differ between the HCC and the non-HCC cases, but the levels at the diagnosis of HCC were higher than at the last follow-up visit in the non-HCC cases (S4 Fig). Serum HBcrAg levels were measured in 152 of the 276 patients in the CH and the LC groups at baseline, which showed that the HBcrAg levels tended to be higher in the HCC cases than in the non-HCC cases (6.7 vs 5.5 log U/ml, *P* = 0.053) (S2 Table). In addition, investigation of HBcrAg levels in 161 of the 276 patients at the diagnosis of HCC or the last follow-up visits revealed that they were significantly higher in the HCC cases than the non-HCC cases (4.4 vs 3.7 log U/ml, *P* = 0.034).

## Discussion

In this study, we compared the long-term risks of developing HCC among CHB patients in the various phases of the disease. This observational study showed that HCC occurred in 3.5% (2/56) and 1.5% (2/134) of the patients in the IT+MA and IC phases at baseline, respectively.

The international guidelines recommend observation rather than treatment during the IT phase [7]. However, no international consensus has been made to define the true IT phase. Our study defined the IT+MA phase as follows: being HBeAg positive, HBV DNA >20,000 IU/ml, and ALT <2×ULN, which is recommended for monitoring and assessment of fibrosis in the APASL guideline. Meanwhile, the APASL guideline defines the IT phase as persistence of HBeAg-positive HBV infection without significant ongoing necro-inflammatory disease of the liver, namely being HBeAg positive, HBV DNA >20,000 IU/ml, and normal ALT level [4]. Liver biopsy is useful for assessment of fibrosis, but repeated liver biopsies for evaluating hepatic inflammation and fibrosis are not easy to carry out because of their risk and/or cost. Therefore, we usually use serum ALT level as a marker of liver damage for evaluating the severity of hepatitis activity [24, 25], but one study showed that approximately 40% of HBeAg positive patients with normal ALT levels had necro-inflammation and fibrosis [26]. Thus, it is difficult to diagnose “true immune-tolerant”. If we define the true IT phase as being HBeAg positive, HBV DNA >20,000 IU/ml, and normal ALT level, 16 of the 56 patients in the IT phase at baseline met the criteria. Of the 16 patients, none developed HCC but 2 progressed to the phase of CH and were treated with NAs. In fact, the IT+MA-HCC case 1 and 2 were not classified as the true IT phase. Building the international consensus to define the true IT phase is desirable.

The IC phase is defined as HBeAg-negative and anti-HBe-positive, HBV DNA <2,000 IU/ml, ALT <ULN [4]. Patients in the IC phase generally have a good prognosis. Long-term follow up of patients in the IC state indicated that the vast majority showed sustained biochemical remission [6, 27]. However, the IC state may occasionally lead to reactivation of hepatitis, cirrhosis and HCC. In the longitudinal study by Tong et al., 145 IC patients with a long follow-up period (mean of 8 years) included 2 who developed HCC but no cases of liver-related death [28]. In our study, the rate of occurrence of HCC was similar to the above study. Another study showed that 20–30% of IC patients may undergo spontaneous reactivation during the follow-up period [29]. However, in our study, only 2 patients assessed as the IC phase at baseline were treated with NAs because of transitioning to the CH phase.

Consistent with many previous studies, our results showed that advanced hepatic fibrosis and older age are risks for patients treated with NAs developing HCC. Importantly, we showed that the cumulative incidence rate of HCC development in CH patients under NA treatment was suppressed to the same levels as the IT+MA and IC groups (Fig 2). The FIB-4 index is a noninvasive marker of hepatic fibrosis, originally developed for patients with chronic hepatitis C [30]. A meta-analysis showed the value of the FIB-4 index in CHB at the point of liver fibrosis [19]. The FIB-4 index was also highly predictive of HCC development in CHB patients treated with entecavir [15]. In our study, FIB-4 index levels were elevated in the CH patients who developed HCC, but decreased in those who did not develop HCC during the follow-up periods. On the other hand, the two IT+MA and one IC patients who developed HCC at the ages of 56, 62 and 73 had higher levels of FIB-4 index during the follow-up and/or at the diagnosis of HCC. Therefore, we suggest considering liver biopsy for patients of the IT+MA or IC status with higher levels of FIB-4, especially if aged more than 40, as the American Association for the Study of Liver Diseases (AASLD) has recommended [5]. The M2BPGi level also has been reported to predict progression of liver disease in chronic hepatitis C [31], non-alcoholic fatty liver disease [32], and CHB [33], and has been shown to be useful for predicting the development of HCC in CHB patients without NA treatment [34], as well as under NA therapy [16]. Our results showed that the CH patients who developed HCC had higher levels of M2BPGi at the diagnosis of HCC. These data suggest that CHB patients with higher levels of FIB-4 index and/or M2BPGi need careful surveillance of developing HCC. In addition, we investigated the relationship between serum HBcrAg levels and development of HCC using limited data of the patients. Previous studies have shown that HBcrAg level >3 log U/mL was an independent risk factor for the occurrence of HCC [35], and patients with persistently elevated HBcrAg levels during ongoing treatment had a higher risk of HCC than similar patients with low HBcrAg levels [36]. Consistent with these results, our study showed that persistent high levels of HBcrAg were associated with development of HCC.

Our study has some important limitations. First, as this is an observational study, the findings were potentially subject to selection bias. For instance, patients in IT+MA group are mostly young and do not need treatment immediately, therefore, the number of patients who visit hospital regularly is smaller than in other groups. Second, treating with NAs was at the discretion of the attending physician. Strictly speaking, there might be cases where NA treatment was started without CH. Third, this study relied on clinical and radiological criteria for diagnosing cirrhosis; therefore, there is the possibility that some patients with advanced hepatic fibrosis were included in the IT+MA, IC, and CH groups. Fourth, this is a single center study. Unlike in many other regions, HBV genotype C, which is associated with a high risk of HCC, is predominant in Japan [37, 38].

In conclusion, our study shows that HCC occurred at a certain rate in patients in the IT+MA and IC groups. Careful follow-up is required for patients in the CH group with higher levels of FIB-4 index and/or M2BPGi because of the high risk of developing HCC.

## Supporting information

S1 Fig Cumulative incidence of hepatocellular carcinoma in patients who had not received interferon treatment among different phases of chronic hepatitis B virus infection. The cumulative incidence curves for hepatocellular carcinoma were estimated using the Kaplan-Meier Method. Abbreviations: IT+MA, immune tolerant + mildly active; IC, inactive carrier; CH, chronic active hepatitis; LC, liver cirrhosis.
(TIF)

S2 Fig Clinical course of the IT+MA-HCC case 1. Abbreviations: IT+MA, immune tolerant + mildly active; HCC, hepatocellular carcinoma; FIB-4, fibrosis-4; HBV, hepatitis B virus; AFP, α-fetoprotein; HBsAg, hepatitis B surface antigen; ALT, alanine aminotransferase; HBeAg, hepatitis B e antigen; HBcrAg, hepatitis B core-related antigen.
(TIF)

S3 Fig Clinical course of the IC-HCC case 2. Abbreviations: IC, inactive carrier; HCC, hepatocellular carcinoma; AFP, α-fetoprotein; HBV, hepatitis B virus; FIB-4, fibrosisi-4; HBeAg, hepatitis B e antigen; HBsAg, hepatitis B surface antigen; HBcrAg, hepatitis B core-related antigen; ALT, alanine aminotransferase.
(TIF)

S4 Fig Transition of M2BPGi in CH group. We depict the transition of M2BPGi levels from the first visit to our hospital to the diagnosis of hepatocellular carcinoma (HCC) or the last follow-up visits of patients who did not develop HCC. Red line and numbers represent mean M2BPGi levels in HCC cases, and those in blue represent in non-HCC cases. M2BPGi levels were compared between HCC and non-HCC groups at the first visit and the diagnosis of HCC or the last follow-up visits using the Mann-Whitney U test. Above the horizonal line represents third quantile, and below the horizonal line represents first quantile. Abbreviations: CH, chronic active hepatitis; HCC, hepatocellular carcinoma.
(TIF)

S1 Table Baseline clinical characteristics of the patients who had not received interferon treatment among different phases of chronic hepatitis B virus infection. (PDF)

S2 Table Baseline clinical characteristics of the patients in CH and LC groups according to the development of hepatocellular carcinoma. (PDF)
