# Supplementary figures and images for: Development of hepatocellular carcinoma from various phases of chronic hepatitis B virus infection
Source: PLoS One. 2021 Dec 28;16(12):e0261878. doi: 10.1371/journal.pone.0261878 (PMC8714106; doi:10.1371/journal.pone.0261878)

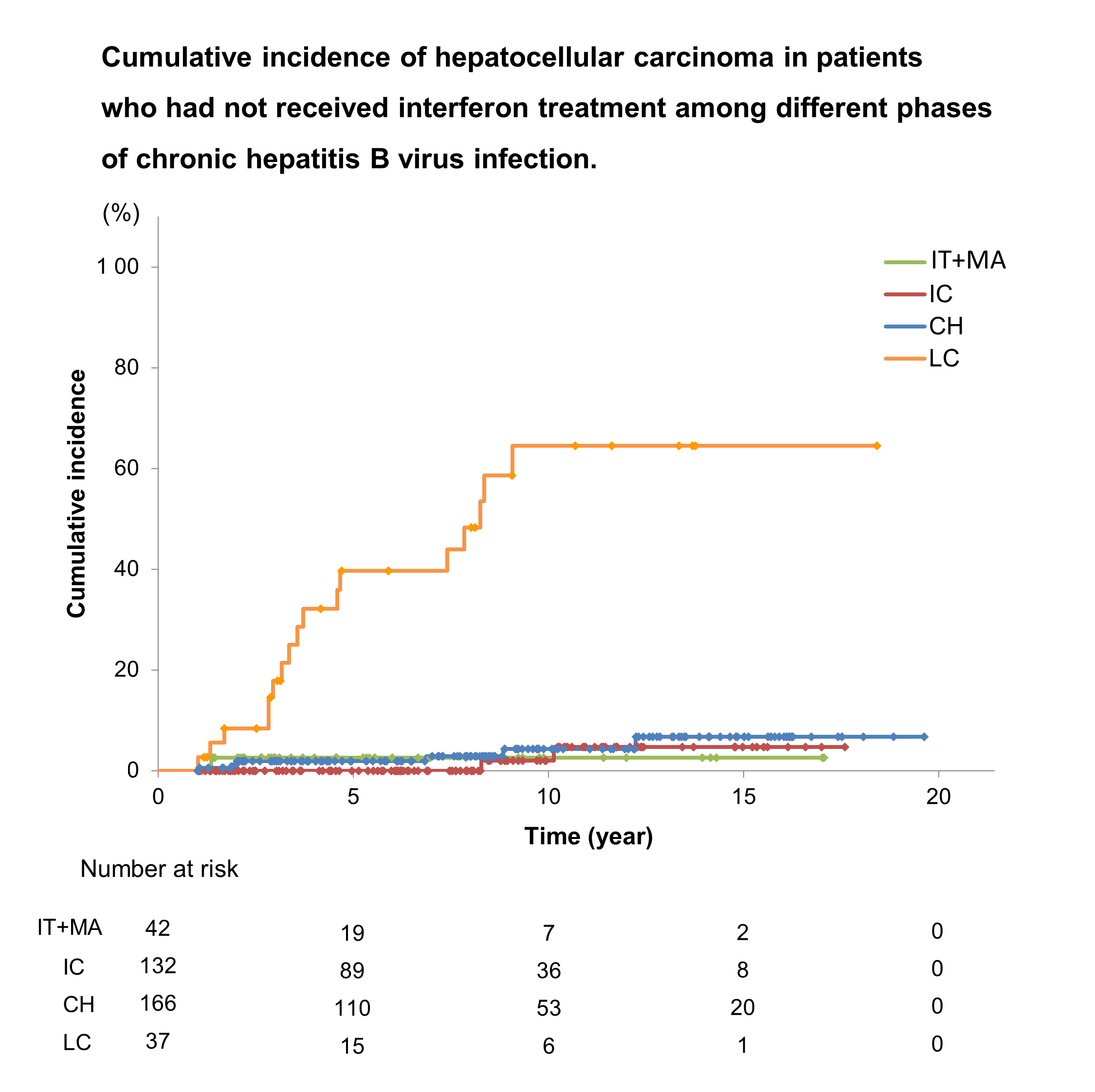

Supplement: S1 Fig — The cumulative incidence curves for hepatocellular carcinoma were estimated using the Kaplan-Meier Method. Abbreviations: IT+MA, immune tolerant + mildly active; IC, inactive carrier; CH, chronic active hepatitis; LC, liver cirrhosis. (TIF) [file pone.0261878.s001.tif]

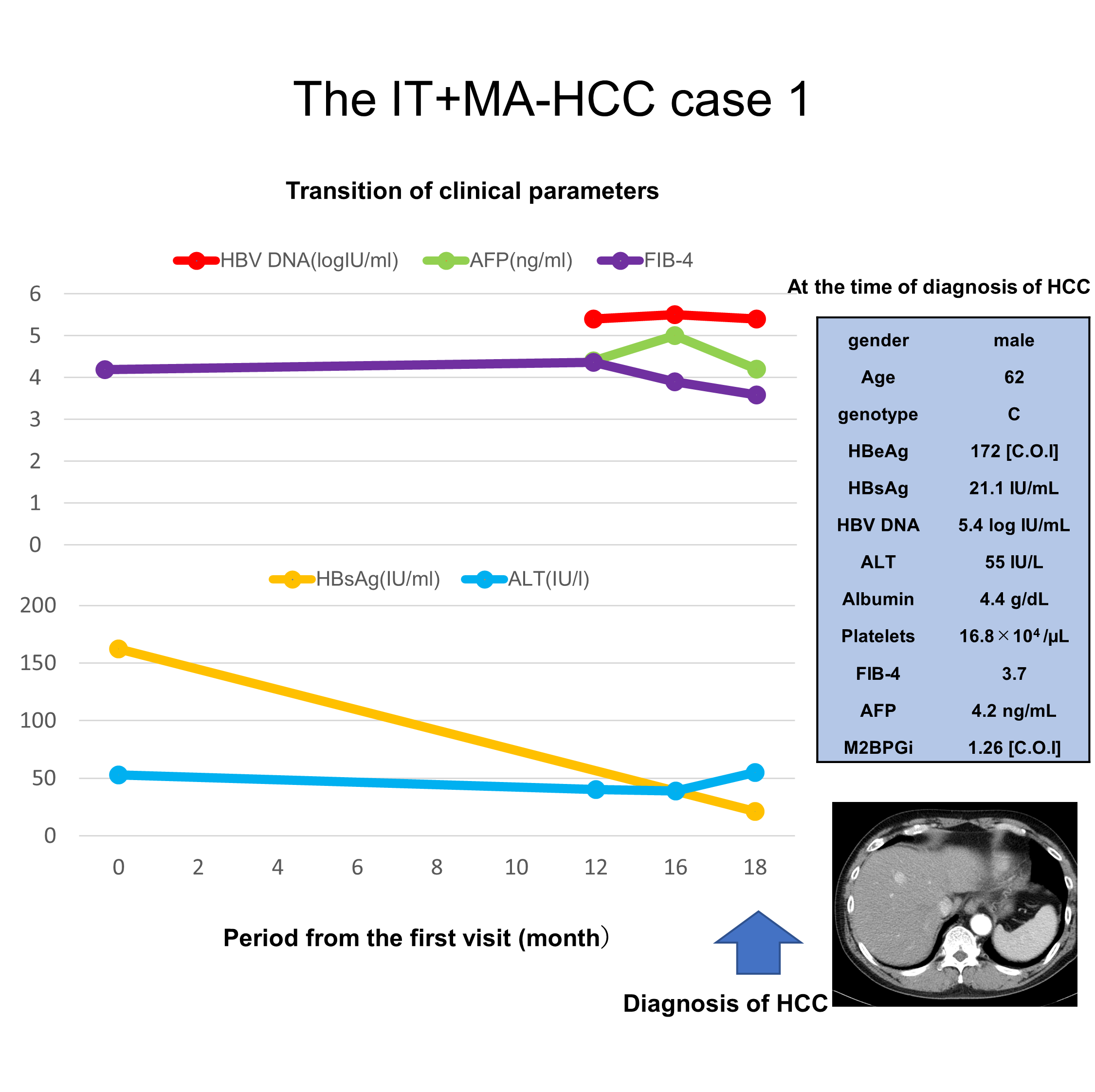

Supplement: S2 Fig — Abbreviations: IT+MA, immune tolerant + mildly active; HCC, hepatocellular carcinoma; FIB-4, fibrosis-4; HBV, hepatitis B virus; AFP, α-fetoprotein; HBsAg, hepatitis B surface antigen; ALT, alanine aminotransferase; HBeAg, hepatitis B e antigen; HBcrAg, hepatitis B core-related antigen. (TIF) [file pone.0261878.s002.tif]

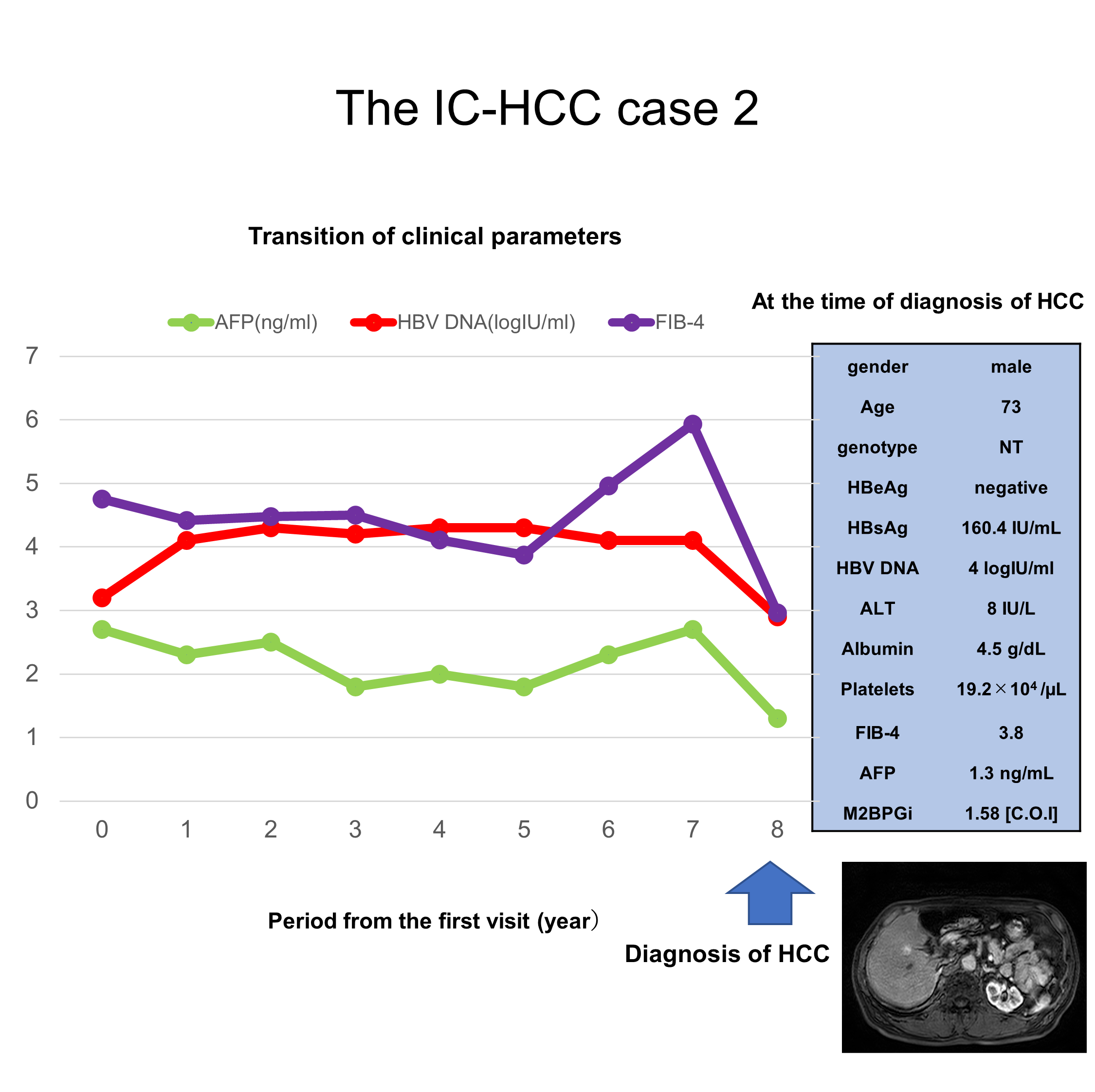

Supplement: S3 Fig — Abbreviations: IC, inactive carrier; HCC, hepatocellular carcinoma; AFP, α-fetoprotein; HBV, hepatitis B virus; FIB-4, fibrosisi-4; HBeAg, hepatitis B e antigen; HBsAg, hepatitis B surface antigen; HBcrAg, hepatitis B core-related antigen; ALT, alanine aminotransferase. (TIF) [file pone.0261878.s003.tif]

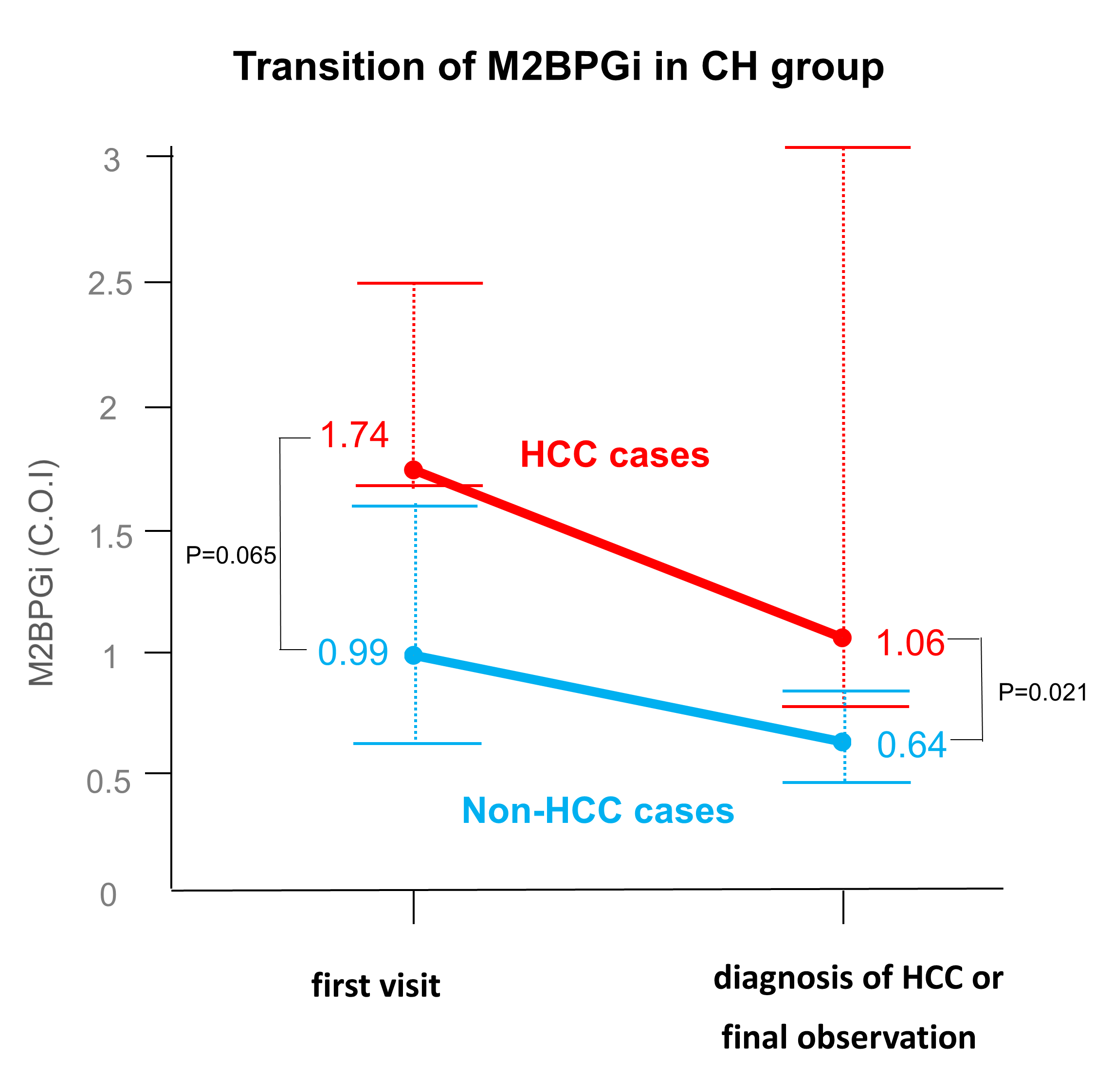

Supplement: S4 Fig — We depict the transition of M2BPGi levels from the first visit to our hospital to the diagnosis of hepatocellular carcinoma (HCC) or the last follow-up visits of patients who did not develop HCC. Red line and numbers represent mean M2BPGi levels in HCC cases, and those in blue represent in non-HCC cases. M2BPGi levels were compared between HCC and non-HCC groups at the first visit and the diagnosis of HCC or the last follow-up visits using the Mann-Whitney U test. Above the horizonal line represents third quantile, and below the horizonal line represents first quantile. Abbreviations: CH, chronic active hepatitis; HCC, hepatocellular carcinoma. (TIF) [file pone.0261878.s004.tif]
