# Supplementary material for: Development of hepatocellular carcinoma from various phases of chronic hepatitis B virus infection
Source: PLoS One. 2021 Dec 28;16(12):e0261878. doi: 10.1371/journal.pone.0261878 (PMC8714106; doi:10.1371/journal.pone.0261878)
Supplement: S1 Table — (PDF) [file pone.0261878.s005.pdf]

**S1 Table. Baseline clinical characteristics of the patients who had not received interferon treatment among different phases of chronic hepatitis B virus infection.**

| | Phases of chronic HBV infection | | | | |
|---|---|---|---|---|---|
| **Characteristic** | **IT+MA** | **IC** | **CH** | **LC** | |
| **Number** | **(n = 42)** | **(n = 132)** | **(n = 166)** | **(n = 37)** | ***P*-value** |
| **Age, years** | **29.5 (26.0 – 37.5)** | **48.0 (37.0 – 59.0)** | **47.0 (40.0 – 54.0)** | **55.0 (46.5 – 60.5)** | **<0.001** |
| **Male gender, n (%)** | **20 (48%)** | **54 (41%)** | **100 (60%)** | **22 (59%)** | **<0.001** |
| **HBV genotype A / B / C / N.D., n** | **2 / 3 / 28 / 9** | **6 / 14 / 48 / 64** | **8 / 15 / 117 / 26** | **0 / 3 / 29 / 5** | **<0.001** |
| **HBV DNA, $log_{10}$IU/ml** | **7.9 (7.1 – 8.1)** | **2.7 (1.9 – 2.9)** | **6.1 (4.7 – 7.3)** | **5.6 (3.4 – 8.2)** | **<0.001** |
| **AST, U/ml** | **27 (19 – 32)** | **21 (18 – 24)** | **62 (35 – 121)** | **67 (44 – 110)** | **<0.001** |
| **ALT, U/ml** | **33 (20 – 41)** | **19 (14 – 23)** | **92 (43 – 202)** | **59 (40 – 132)** | **<0.001** |
| **Albumin, g/dl** | **4.4 (4.0 – 46)** | **4.5 (4.3 – 4.7)** | **4.3 (4.1 – 4.6)** | **3.7 (3.1 – 4.3)** | **<0.001** |
| **Total bilirubin, mg/dl** | **0.6 (0.5 – 0.8)** | **0.7 (0.6 – 0.9)** | **0.8 (0.6 – 1.0)** | **1.0 (0.8 – 2.0)** | **<0.001** |
| **Platelets, $\times 10^4$/dl** | **19.8 (18.0 – 24.5)** | **21.0 (18.1 – 23.8)** | **17.5 (14.4 – 21.4)** | **8.5 (7.0 – 11.9)** | **<0.001** |
| **FIB-4 index** | **0.66 (0.53 – 0.95)** | **1.12 (0.79 – 1.43)** | **1.78 (1.19 – 2.76)** | **4.76 (3.47 – 8.98)** | **<0.001** |
| **HBsAg, IU/ml** | **2000 (2000 – 4545)** | **1640 (470 – 2000)** | **2580 (1342 – 8943)** | **1460 (474 – 2000)** | **<0.001** |
| **HBeAg positive, n (%)** | **42 (100%)** | **0 (0%)** | **93 (56%)** | **19 (51%)** | **<0.001** |
| **Duration of follow up period, month** | **52.0 (28.1 – 108.2)** | **90.9 (43.6 – 126.6)** | **97.3 (49.0 – 149.6)** | **97.6 (38.0 – 141.4)** | **<0.001** |

Data from all patient were expressed as numbers for categorical data and medians (first–third quartiles) for noncategorical data.

Categorical variables were compared between groups by the Kruskal-Wallis test, and noncategorical variables were compared using the chi-square test.

Abbreviations: HBV, hepatitis B virus; IT+MA, immune tolerant + mildly active; IC, inactive carrier; CH, chronic active hepatitis; LC, liver cirrhosis; N.D, not determined; AST, aspartate transaminase; ALT, alanine transaminase; FIB-4, fibrosis-4; HBsAg, hepatitis B surface antigen; HBeAg, hepatitis B e antigen; IFN, interferon
