# Supplementary material for: Development of hepatocellular carcinoma from various phases of chronic hepatitis B virus infection
Source: PLoS One. 2021 Dec 28;16(12):e0261878. doi: 10.1371/journal.pone.0261878 (PMC8714106; doi:10.1371/journal.pone.0261878)
Supplement: S2 Table — (PDF) [file pone.0261878.s006.pdf]

S2 Table. Baseline clinical characteristics of the patients in CH and LC groups according to the development of hepatocellular carcinoma.

Characteristic	HCC	non-HCC	
**Number**	**(n = 29)**	**(n = 247)**	***P*-value**
**Age, years**	**57 (50 – 66)**	**44 (37 – 52)**	**<0.001**
**Male gender, n (%)**	**17 (70%)**	**161 (65%)**	**N.S.**
**CH / LC**	**9 / 20**	**228 / 19**	**<0.001**
**HBV DNA, log_{10}IU/ml**	**6.1 (4.9 – 6.9)**	**6.1 (4.7 – 7.3)**	**N.S.**
**AST, U/ml**	**67 (46 – 161)**	**66 (37 – 126)**	**N.S.**
**ALT, U/ml**	**68 (42 – 150)**	**96 (45 – 223)**	**N.S.**
**Albumin, g/dl**	**3.8 (3.1 – 4.4)**	**4.3 (4.1 – 4.6)**	**0.002**
**Total bilirubin, mg/dl**	**1.0 (0.7 – 1.6)**	**0.8 (0.6 – 1.0)**	**0.013**
**Platelets, $\times 10^4$/dl**	**8.9 (5.6 – 13.8)**	**17.0 (14.1 – 21.3)**	**<0.001**
**M2BPGi**	**7.62 (2.35 – 13.61)**	**1.08 (0.70 – 1.91)**	**<0.001**
**FIB-4 index**	**5.35 (3.53 – 11.52)**	**1.72 (1.19 – 2.82)**	**<0.001**
**HBsAg, IU/ml**	**1493 (1170 – 2472)**	**2588 (1175 – 9161)**	**0.028**
**AFP, ng/ml**	**18.9 (5.6 – 48.3)**	**4.4 (2.7 – 9.3)**	**<0.001**
**HBcrAg, log U/ml**	**6.7 (5.5 – 6.8)**	**5.5 (4.3 – 6.8)**	**0.053**

Data from all patient were expressed as numbers for categorical data and medians (first–third quartiles) for noncategorical data. Categorical variables were compared between groups by the Kruskal-Wallis test, and noncategorical variables were compared using the chi-square test.

Abbreviations: CH, chronic active hepatitis; LC, liver cirrhosis; HCC, hepatocellular carcinoma; HBV, hepatitis B virus; AST, aspartate transaminase; ALT, alanine transaminase; FIB-4, fibrosis-4; HBsAg, hepatitis B surface antigen; AFP, α-fetoprotein; HBcrAg, hepatitis B core-related antigen.
